# Development and cross-validation of predictive equations for fat-free mass estimation by bioelectrical impedance analysis in Brazilian subjects with overweight and obesity

**DOI:** 10.3389/fnut.2025.1499752

**Published:** 2025-01-20

**Authors:** Kalina Veruska Da Silva Bezerra Masset, Analiza M. Silva, Gerson Ferrari, Breno Guilherme De Araújo Tinoco Cabral, Paulo Moreira Silva Dantas, Roberto Fernandes Da Costa

**Affiliations:** ^1^Post-graduate Program in Pharmaceutical Sciences, Health Sciences Center, Federal University of Rio Grande do Norte, Natal, Brazil; ^2^Exercise and Health Laboratory, CIPER, Faculdade Motricidade Humana, Universidade de Lisboa, Lisbon, Portugal; ^3^Department of Movement Sciences and Sports Training, School of Sport Sciences, The University of Jordan, Amman, Jordan; ^4^Escuela de Ciencias de la Actividad Física, el Deporte y la Salud, Universidad de Santiago de Chile (USACH), Santiago, Chile; ^5^Faculty of Health Sciences, Universidad Autónoma de Chile, Providencia, Chile; ^6^Department of Physical Education, Health Sciences Center, Federal University of Rio Grande do Norte, Natal, Brazil

**Keywords:** body composition, resistance index, absorptiometry, adults, mathematical models

## Abstract

**Introduction:**

Obesity is a public health problem worldwide, and body composition assessment is a very important diagnostic tool. Bioelectrical Impedance Analysis (BIA) is a fast, non-invasive, relatively low-cost, and user-friendly technique; however, to obtain greater validity of the estimates, the predictive equations used must be population specific. Thus, the objectives of this study were: (1) to test the validity of four BIA equations used for fat-free mass (FFM) estimation and one model for fat mass (FM) estimation in adults with overweight or obesity; (2) develop and cross-validate new equations to estimate FFM to adults with overweight or obesity, and specific for those with obesity.

**Methods:**

The non-probabilistic sample included 269 individuals, 53.2% with overweight and 46.8% with obesity, aged 18–79 years, randomly divided into two groups: development (*n* = 178) and cross-validation (*n* = 91), stratified by sex and classification as overweight or obese. The criterion technique was dual-energy-x-ray absorptiometry (DXA), whereas a tetrapolar single-frequency BIA equipment was used as the alternative method. Paired t-test, multiple regression, concordance correlation coefficient, and Bland–Altman analysis were used.

**Results:**

Most existing equations were not valid and new equations were derived: (1) for individuals with overweight or obesity: CCC = 0.982; r^2^ = 0.95; standard error of estimate (SEE) = 2.50 kg; limits of agreement (LOA) = -5.0 to 4.8; and (2) specific for individuals with obesity: CCC = 0.968; r^2^ = 0.94; SEE = 2.53 kg; LOA = -5.3 to 5.2. No FFM differences were observed between the new models and the reference method (*p* > 0.05).

**Conclusion:**

The new proposed models provide valid options to estimate FFM in an adult population with overweight/obesity.

## Introduction

Over the last 40 years, the prevalence of obesity has been increasing around the world; it now constitutes one of the most important public health issues, mainly due to its association with other chronic non-communicable diseases and the related increases in morbidity and mortality, which cause high costs for health systems (1–3). It has long been known that overweight, the range between normal weight and obesity, can also increase risks for adverse health outcomes (4–6).

Overweight and obesity are defined as presenting a high body mass index (BMI) (≥25 kg/m^2^ and ≥ 30 kg/m^2^, respectively), according to the World Health Organization (WHO) criteria (7). BMI is very useful for carrying out epidemiological studies, but for individual clinical diagnosis it can present serious biases as it does not allow clinicians to identify whether excess weight is due to fat mass or lean body mass, suggesting the need to apply body composition assessment techniques (8).

In clinical and field settings, one of the most commonly used ways to assess body composition is bioelectrical impedance analysis (BIA). It is a fast, simple, and relatively low-cost technique that provides estimates of fat-free mass (FFM) and fat mass (FM) based on mathematical models (9–12). The equipment used in BIA measures the resistance (R) and reactance (Xc) of the body to the passage of a low amperage electric current, which can be single-(50 kHz) or multi-frequency. The body is modeled as a cylindrical-shaped ionic conductor in BIA-based assessment, with R reflecting the resistivity of body water, whereas Xc is due to the capacitive nature of cell membranes (13). These raw BIA measures, in combination with other variables such as height, body mass, sex and age, are used to develop specific predictive equations for the studied population (13, 14). If these equations are used to assess the body composition of populations with characteristics different from the original population, then the results may be inconsistent, indicating that they cannot be generalized to various population groups (13). Indeed, different population groups may show different amounts of water in the FFM depending on sex, age and ethnicity, suggesting the need for specific equations addressing these differences (15).

Few equations have been developed and validated to evaluate FFM in individuals with overweight or obesity using BIA, among them there are two that were produced a long time ago using hydrostatic weighing as a standard technique, which are still frequently used in clinical practice (16, 17). Subsequently, other generalized equations were developed to estimate FFM, using DXA (18) or air displacement plethysmography (ADP) (19) as reference methods. In another study, an equation for BIA was developed using a four-compartment model, which is the reference method used to assess FM, but the equation was developed only for older Hispanic adults (20). In Brazil, an equation was developed to estimate body fat, using ADP as the gold standard method (21); however, applied only to severely obese individuals, which may reduce the validity of the results for the most prevalent obesity categories, that is, classes I and II.

The use of predictive equations in subjects with characteristics different from those of the group from which the equations originate is questionable, as it increases the risk of bias in the results (22). Even so, many professionals settle for using these inadequate mathematical models due to the lack of specific equations for the group they intend to evaluate. In addition, many BIA devices do not describe the equations that are available in their software, nor for which population groups these were developed and validated (9, 12).

Our hypothesis is that the use of predictive equations such as those of Segal et al. (17) and Gray et al. (16), or other equations developed for populations different from ours, are not valid for adults with overweight or obesity in the Brazilian population; therefore, the development and cross-validation of specific equations for subjects with these characteristics may overcome the inaccuracies of previous models given the marked morphological differences related to ethnic miscegenation (23).

Thus, the objectives of the present study were as follows: (1) to test the validity of four BIA equations used for FFM estimation and one model used for FM prediction in adults with overweight or obesity; and (2) to develop and cross-validate new equations to use for estimating fat-free mass in adults with overweight or obesity, and specificity for those with obesity, in the Brazilian population.

## Methods

This is a descriptive cross-sectional study to test the validity of existing equations, in addition to the development and cross-validation of regression equations to estimate body composition, in volunteers living with overweight or obesity, between January 2018 and April 2019, in the city of Natal. It was conducted according to the guidelines laid down in the Declaration of Helsinki, and all procedures involving human subjects/patients were approved by the Research Ethics Committee of the University Hospital Onofre Lopes - HUOL/UFRN, Natal-RN (# 34804414.7.0000.5292). All participants in this study gave informed consent at the time of recruitment by signing a written informed consent form.

### Sample

**Figure 1:**
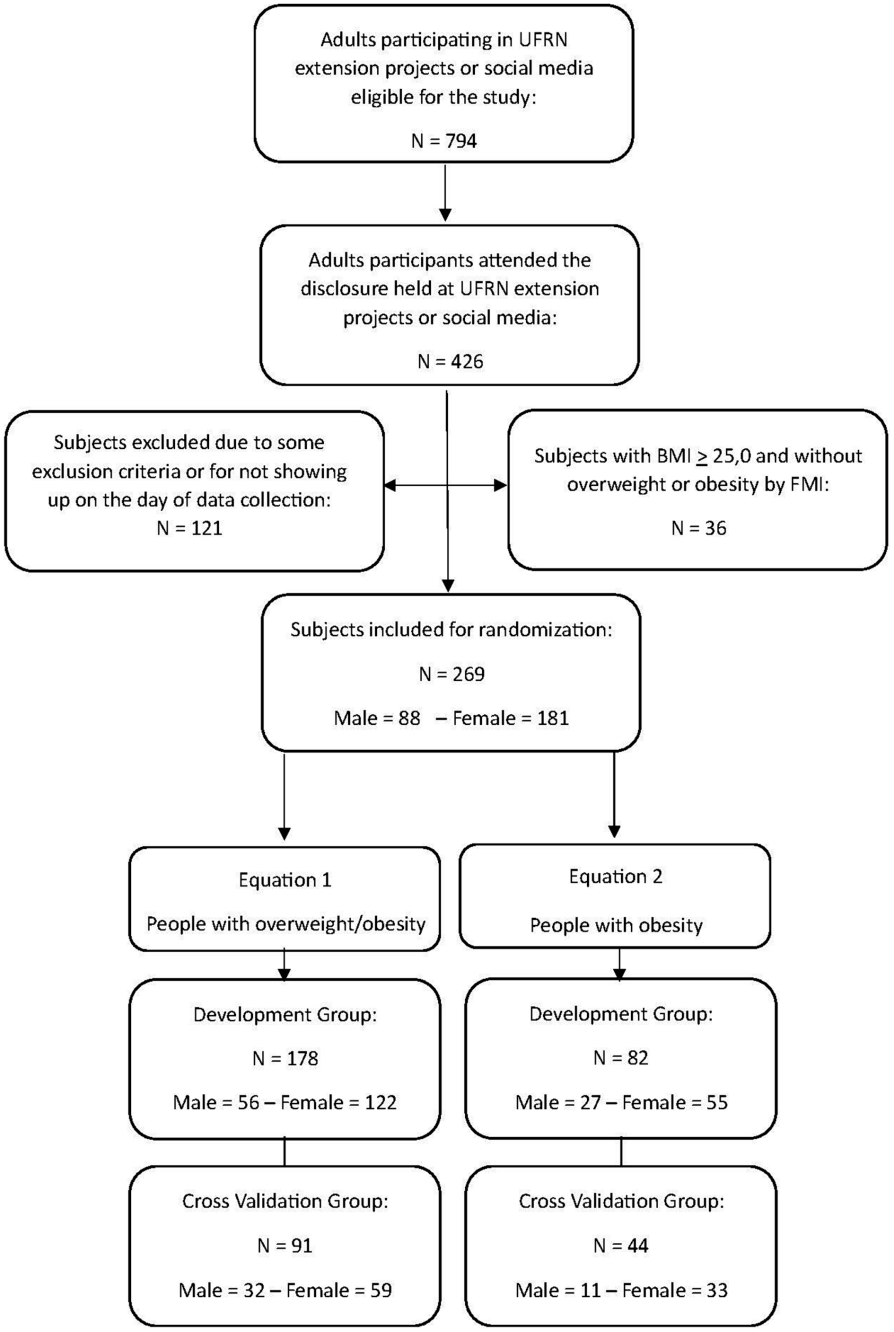
Flowchart of the study protocol, showing the subjects eligible for the study, those who met the criteria and attended the assessments, randomly selected for the development group and cross-validation group.

The recruited study population consisted of 269 subjects (181 women), aged 18–79 years and from the northeast region of Brazil, who were recruited through the dissemination of invitations to participate by students participating in university extension projects at the Physical Education Department of the Federal University of Rio Grande do Norte (UFRN), through nomination by participants, or through social media (Figure 1). After their inclusion in the study, the sample was randomly divided into two groups, namely, the development of a predictive equation of FFM (*n* = 178) and cross-validation (*n* = 91), stratified by sex and fat mass index (FMI) status (24), overweight or obesity. For the sample size calculation, using FFM as a primary outcome, we considered a medium to small effect size (0.10) with six predictors (independent variables), with a type I error of 5% and a power of 95%. Using these parameters, a total of 132 participants were required.

The inclusion criteria allowed recruitment of subjects of both sexes who were aged 18–79 years, had a BMI greater than or equal to 25 kg/m^2^, were without chronic diseases, and were not taking medication that could interfere with normal fluid-electrolyte balance and potentially affect body composition results. The exclusion criteria comprised pregnancy; hypovolemic or hypervolemic conditions, including diet, diuretic, or corticoid use; edema; any physical disability; or use of a prosthesis that could alter the results of the body composition assessment. Furthermore, after assessing fat mass by DXA, subjects who presented a BMI > 25.0 kg/m^2^ but were not classified as overweight or obese by the FMI were also excluded from the sample.

All data collection was conducted during a single laboratory visit by each participant, after overnight fasting, which involved the collection of anthropometric measurements followed by BIA assessment and then DXA assessment.

### Anthropometric measurements

Anthropometric measurements were performed by a single physical education professional, who was properly trained in accordance with international recommendations (25). Body mass was measured using a digital scale with 0.1 kg resolution from Sanny^®^, model BL200PP (American Medical do Brasil, São Bernardo do Campo, Brazil), while participants were barefoot and wearing light clothes. In addition, all jewelry and metal objects were removed for this and all subsequent measurements. Height was measured using a stadiometer from Sanny^®^ with a resolution of 0.1 cm, Caprice model (American Medical do Brasil, São Bernardo do Campo, Brazil), with the participants barefoot and in an orthostatic position. BMI was calculated by dividing body mass (kg) by the square of height (m) and was classified as overweight or obese using cutoff points proposed by the WHO (7).

### Bioelectrical impedance analysis

The BIA assessment for the determination of resistance (R), reactance (Xc) and phase angle (PhA) was conducted with single-frequency tetrapolar equipment (50 kHz) at a current of 800 μA, using equipment from Sanny^®^, BIA1010 model (American Medical do Brasil, São Bernardo do Campo, Brazil). As this equipment does not provide the phase angle, we calculated it using the formula PhA = tangent arc Xc/R x 180°/ *π*. The resistance index was calculated as the ratio between height squared by resistance (Ht^2^/R). The BIA equipment validity measurement was periodically taken using an electrical resistor and capacitor; calibration values were considered normal if the R was not higher than 500 ± 5 ohm (*Ω*) and Xc was not higher than 52 ± 0.5 Ω, according to the manufacturer’s instructions. Sanny’s bioimpedance equipment was chosen because it is the only one manufactured in Brazil, which allows easy access in this country and relatively low operating costs.

Verification of the quality of the measures obtained by the equipment was carried out in a previous study with 46 women from the northeast region of Brazil. The results obtained included coefficient of variation (CV) values of 0.17 and 0.72% for R and Xc, respectively, and a technical error of measurement (TEM) of 0.76 *Ω* (0.22%) and 0.35 Ω (0.92%) for R and Xc, respectively (9).

Participants were evaluated after lying down for 10 min in the supine position on a non-conductive stretcher, with both arms and legs abducted 30° from the midline of the body. To avoid short-circuiting of equipment in participants with obesity, a foam device was used between the lower limbs. The skin was cleaned with 70% alcohol before placing the electrodes, which were positioned on the dorsal surface of the wrist, hand, ankle, and foot, in the right hemi body. Participants were asked to fast overnight before the assessment, not to perform any strenuous physical exercise in the previous 24 h, and not to consume alcohol in the previous 48 h. In addition, they were asked to empty their bladder 30 min before the assessment. The resistance index (Ht^2^/R) was calculated by dividing the square of height (m) by R (*Ω*).

### Dual energy X-ray absorptiometry

Dual energy X-ray absorptiometry was performed with Lunar Prodigy equipment, NRL 41990 model (GE Lunar, Madison, WI, United States) by a laboratory technician experienced in radiological evaluation. The scan was conducted with the participant lying in the supine position along the longitudinal axis of the midline of the table. The participant’s feet were positioned together and stuck at the level of the fingers to immobilize the legs, while the hands were held in the prone position within the scanning region of the equipment. The participant remained still during the digitalization process. Measurements were performed following the recommendations proposed in the literature (26), and body composition was determined using enCoreTM 2011 version 13.6 software (GE Health Lunar). As described in a study that developed equations for estimating FFM in a sample of 396 male Brazilian Army cadets, aged 17–24 years, the CVs for FM, bone mineral content (BMC) and lean soft tissue (LST) using the current equipment were 0.74, 0.28 and 0.26%, respectively (23). The TEMs were 0.25, 0.02 and 0.25 kg for FM, BMC and LST, respectively. The authors reported that the TEM was calculated based on the test–retest procedure that was carried out with 23 subjects from their study population (23). The FFM was obtained by calculating the sum of BMC and LST (FFM = BMC + LST).

### Statistical analysis

The Kolmogorov–Smirnov test was applied to test whether data measures followed a normal distribution. Descriptive statistics were presented using the mean and standard deviation for variables with a normal distribution or the median and interquartile range for variables with a non-normal distribution. Comparisons between groups were performed using the Student’s t test for independent samples or the Mann–Whitney U test. Based on univariate analysis, potential predictor variables were chosen that showed a statistical association with the variable predicted by simple linear regression (*p* ≤ 0.2). Then, forward stepwise regression analysis was performed using FFM obtained by DXA as the dependent variable, with age, sex, body mass, height, R, Xc, PhA, R index, used as possible independent variables. During model development, the normality of the residuals and the homogeneity of the variance were tested. An optimum model was considered the one with the highest r^2^ value (≥0.80), lowest pure error (PE) value (≤5.0), and the Mallows’s Cp value closest to the number of regressors. Significance at *p* < 0.05 was established as a criterion for inclusion of a predictor, whereas exclusion criteria were established at *p* > 0.1. If more than one variable remained in the model, then to assess multicollinearity, a variance inflation factor (VIF) and tolerance (reciprocal of VIF) were calculated for each independent variable, with a VIF < 10 or a tolerance greater than 0.1 used as a threshold to exclude the variable (11, 27). To verify the validity of the proposed equation, the average of estimated results was compared with the average of results measured in DXA by the paired Student’s t test. In addition, Pearson’s correlation coefficient (r), coefficient of determination (r^2^) and standard error of estimate (SEE) were calculated.

For cross-validation of the equations proposed in this study, a multiple regression analysis was performed. In addition, the approach proposed by Lawrence and Lin was used for concordance correlation coefficient (CCC) analysis to verify the validity (Cb) and accuracy (r) of estimated FFM values compared to measured FFM values in the cross-validation group (28). To interpret the CCC results, we accepted values that are considered substantial (0.95 to 0.99) or almost perfect (> 0.99) (29), and r values that are considered very strong (>0.90) (30).

In turn, the accuracy of the new BIA equations was evaluated using PE, and Bland–Altman plots were used to verify bias and concordance between FFM measurements and estimates (31). This method involves plotting the differences between two measurements (i.e., FFM from the DXA minus BIA-predicted FFM) on the y-axis against their means on the x-axis. Each data point on the plot represents the magnitude of the difference between the two measurements. The plot includes horizontal lines representing the mean difference between the two measurements and the limits of agreement (LOA), which are calculated as the mean difference ± 1.96 times the standard deviation of the differences. These lines provide a visual representation of the average discrepancy between the two methods and the range within which most differences fall. Additionally, the same procedures were used to test the validity of the other five equations proposed for estimating FFM, which were either generalized or specific for adults with overweight or obesity (16–19, 21). Analyses were carried out with the statistical package SPSS v.20.0 (SPSS Inc., IBM Corp., Armonk, New York, NY, United States) and MedCalc version 12.5.0. Statistical significance was defined as *p <* 0.05 for all tests.

## Results

Table 1 presents the physical characteristics and body composition variables for the developmental and cross-validation groups, as well as for the whole sample; no differences were observed between the two whole groups (i.e., developmental and cross-validation; *p* > 0.05). Table 2 presents the five equations analyzed in the present study and the characteristics of their samples.

**Table 1:** Descriptive characteristics of the total sample and stratified by sex, including demographic and body composition variables to the groups of development and cross-validation (mean + sd).

	Development group			Cross-validation group		
	Male (*n* = 56)	Female (*n* = 122)	Whole sample (*n* = 178)	Male (*n* = 32)	Female (*n* = 59)	Whole sample (*n* = 91)
Age (yrs)	40.3 ± 12.8	52.1 ± 17.2	48.4 ± 16.8	42.9 ± 9.4	51.5 ± 17.9	48.5 ± 15.2
Nutritional Status - *n* (%)						
Overweight	29 (51.8%)	67 (54.9%)	96 (53.9%)	21 (65.6%)	26 (44.1%)	47 (51.6%)
Obesity	27 (48.2%)	55 (45.1%)	82 (46.1%)	11 (34.4%)	33 (55.9%)	44 (48.4%)
Body mass (kg)	91.0 ± 12.3	76.5 ± 11.2	81.1 ± 13.3	87.2 ± 10.1	76.2 ± 10.7	80.1 ± 11.7
Height (cm)	175.4 ± 7.6	159.6 ± 8.1	164.6 ± 10.8	173.4 ± 6.8	158.3 ± 5.2	163.1 ± 9.3
BMI (kg/m^2^)	29.5 ± 3.0	30.1 ± 4.3	29.9 ± 4.0	29.0 ± 2.8	30.4 ± 4.2	29.9 ± 3.8
FMI (kg/m^2^)	9.1 ± 2.3	13.3 ± 3.2	11.9 ± 3.6	8.5 ± 1.8	13.3 ± 3.2	11.9 ± 3.6
Body mass – DXA (kg)	91.2 ± 13.1	76.8 ± 10.9	81.3 ± 12.9	87.5 ± 10.6	76.6 ± 11.3	80.4 ± 11.1
FM (kg) – DXA	28.0 ± 7.8	32.6 ± 7.8	31.9 ± 8.2	25.4 ± 5.5	33.3 ± 7.9	30.5 ± 8.1
FM (%) – DXA	30.5 ± 5.2	43.6 ± 5.2	39.5 ± 8.0	29.0 ± 4.0	43.3 ± 5.2	38.3 ± 8.4
FFM (kg) – DXA	63.0 ± 7.5	42.9 ± 5.7	49.2 ± 11.3	61.8 ± 6.6	42.9 ± 5.0	49.5 ± 10.6
BMC (kg) – DXA	3.1 ± 0.4	2.5 ± 0.3	2.8 ± 0.6	3.2 ± 0.5	2.4 ± 0.3	2.9 ± 0.5
LSTM (kg) – DXA	59.2 ± 7.7	40.9 ± 6.7	47,1 ± 8.0	58.3 ± 7.9	40.3 ± 7.3	46,8 ± 8.2
Resistance (*Ω*)	448.9 ± 50.3	547.0 ± 75.3	516.1 ± 82.1	433.7 ± 52.9	542.6 ± 71.0	504.3 ± 83.3
Reactance (*Ω*)	55.2 ± 8.2	57.4 ± 9.6	56.7 ± 9.2	54.0 ± 7.3	58.6 ± 11.1	57.0 ± 10.1
Phase angle (°)	7.0 ± 0.9	6.0 ± 0.8	6.3 ± 0.9	7.1 ± 0.7	6.2 ± 0.8	6.5 ± 0.9
Resistance Index (Ht^2^/R)	69.5 ± 9.8	47.5 ± 7.7	55.4 ± 13.2	70.6 ± 11.0	47.0 ± 7.1	55.3 ± 14.2

BMI, body mass index; FMI, fat mass index; FM, fat mass; FFM, fat-free mass; BMC, bone mineral content; LSTM, lean soft tissue mass; Ht, height.

**Table 2:** Analyzed equations and respective characteristics of their samples.

Gray et al. (16) - 25 men and 62 women; aged 19 to 74 years; BMI - 19.6 ± 53.3 kg/m^2^ Male FFM = 39.83 + (0.00139 Ht^2^) - (0.0801 R) + (0.187 BM) Female FFM = 20.387 + (0.00151 Ht^2^) - (0.0344 R) + (0.14 BM) - (0.158 Age)
Kyle et al. (18) - 202 men and 141 women; aged 20 to 94 years; BMI - 17.0 to 33.8 kg/m^2^ FFM = −4.104 + (0.518 Ht^2^/R) 1 (0.231 BM) + (0.130 Xc) + (4.229 sex: men = 1, women = 0)
Macias et al. (19) - 73 men and 82 women; aged 20 to 50 years; BMI - 17.8 to 35.5 kg/m^2^ FFM = −2.4658 + 0.7374 (Ht^2^ /R) + (0.1763 BM) - (0.1773 Age) + (0.1198 Xc)
Segal et al. (17) - 597 men and 175 women; aged 17 to 62 years Male - BF ≥ 20% LBM = 14.52435 + (0.0008858 Ht^2^) - (0.02999 R) + (0.42688 BM) - (0.07002 Age) Female - BF ≥ 30% LBM = 9.37938 + (0.00091186 Ht^2^) - (0.01466 R) + (0.29990 BM) - (0.07012 Age)
Horie et al. (21) - 36 men and 83 women; aged 18 to 62 years; BMI - 34.4 to 59.6 kg/m^2^ FM = 23.25 + (0.13 Age) + (1.0 BM) + (0.09 R_50_) - (0.80 Ht)

BM, body mass; BF, body fat; FM, fat mass; FFM, fat-free mass; LBM, lean body mass; Ht, height; R, resistance; Xc, reactance; Ht^2^/R, resistance index.

**Figure 2:**
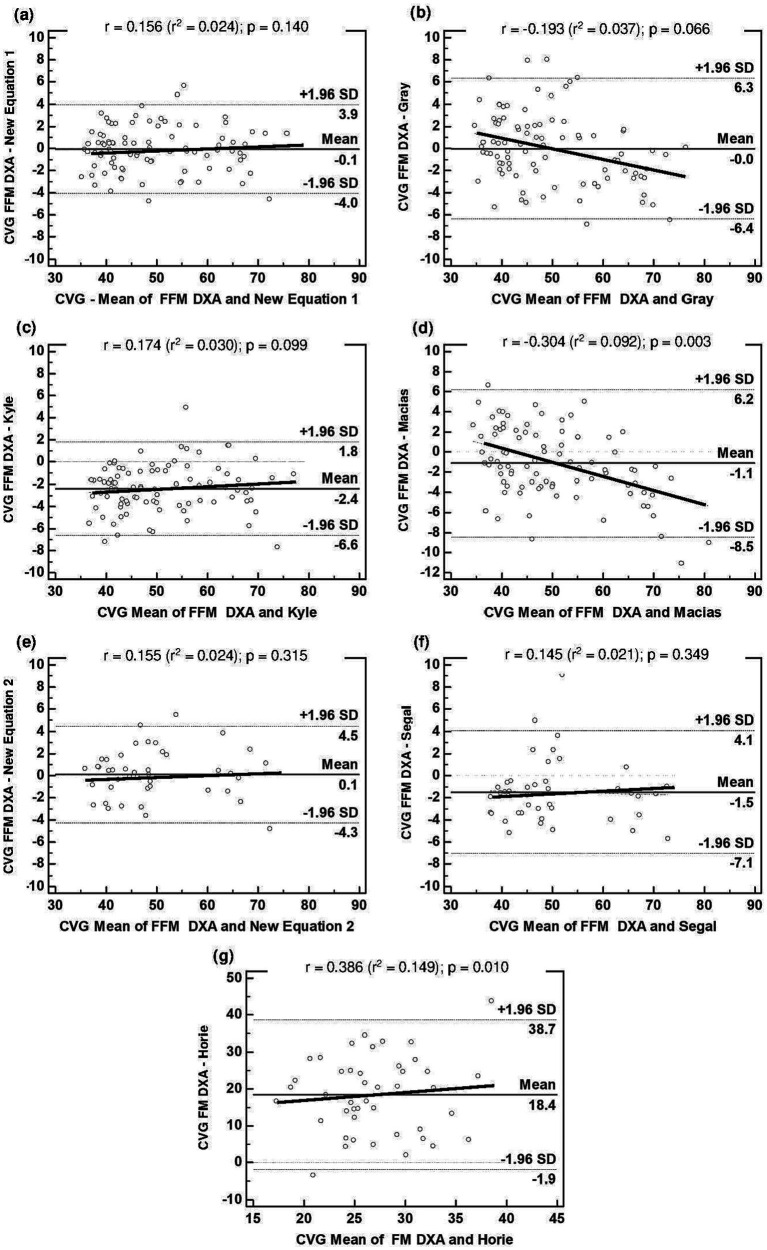
Bland–Altman plots for the concordance limits between values of CVG determined by the reference method (DXA) and equations: **(A)** FFM derived in this study for people with overweight or obesity (Equation 1); **(B)** FFM estimated by Gray et al. (16); **(C)** FFM estimated by Kyle et al. (18); **(D)** FFM estimated by Macias et al. (19); **(E)** FFM derived in this study for people with obesity (Equation 2); **(F)** FFM estimated by Segal et al. (17); **(G)** FM estimated by Horie et al. (21). The middle solid line represents the fixed bias. The dashed lines represent 95% limits of agreement. The proportional bias is represented by the slopping line. BIA, bioelectrical impedance analysis; CVG, Cross Validation Group; DXA, dual-energy X-ray absorptiometry; Equation 1, first new regression equation developed to people with overweight or obesity; Equation 2, second new regression equation developed to people with obesity; FFM, fat-free mass; FM, fat mass; SD, standard deviation.

Analysis of the validity of the three generalized equations (Table 3) and the two specific equations (Table 4), which was performed with the cross-validation group, showed that only the equation developed by Gray et al. (16) met the validity criteria, but that this was with high LOA. None of the other equations that were tested demonstrated validity for the evaluation of our sample, with each presenting a significant difference when compared to the reference method (*p* < 0.001). Furthermore, an association was found between the mean and difference of the BIA and DXA methods (*p* < 0.05) for the equations developed by Macias et al. (19) and Horie et al. (21). The mean difference in the Bland–Altman plot was not different from zero only for the equation by Gray et al. (16) (*p* > 0.05). All equations showed high LOA, indicating low agreement with the reference method (Figure 2). These results justify the need to develop and validate specific equations for our population of overweight or obese people.

**Table 3:** Cross-validation of FFM (kg) predictive new equation 1, and validation of other published generalized equations, in the Cross-validation Group (*n* = 91).

	FFM (kg)	CCC Analysis						
		*p*-value*	CCC	*ρ*	C_b_	r^2^	PE (kg)	SEE (kg)
DXA	49.5 ± 10.3							
New equation 1	49.6 ± 10.6	0.814	0.9817	0.9817	1.0000	0.964	2.19	2.04
Gray et al. (16)	47.6 ± 11.7	0.952	0.9577	0.9617	0.9959	0.925	3.48	2.93
Kyle et al. (18)	51.9 ± 10.4	<0.001	0.9544	0.9795	0.9744	0.958	3.46	2.16
Macias et al. (19)	50.7 ± 12.2	0.005	0.9416	0.9547	0.9863	0.912	4.11	3.18

New equation 1 = persons with overweight or obesity; FFM, fat-free mass; CCC, Concordance Correlation Coefficient; ρ, accuracy; C_b_, validity; PE, pure error; SEE, Standard Error of Estimate. ^*^Differences between predictive equations and reference method by paired *t* test.

**Table 4:** Cross-validation of FFM (kg) and FM (%) predictive new equation, and validation of other published equations, in the cross-validation group (*n* = 44).

	FFM (kg)		CCC Analysis					
		*p*-value*	CCC	ρ	C_b_	r^2^	PE (kg)	SEE (kg)
DXA	49.3 ± 10.3							
New equation 2	49.1 ± 10.3	0.710	0.9764	0.9765	0.9999	0.953	3.33	2.25
Segal et al. (17)	50.7 ± 10.4	0.001	0.9523	0.9624	0.9895	0.925	4.80	2.84

	FM (%)	*p*-value*	CCC	ρ	C_b_	r^2^	PE (kg)	SEE (kg)
DXA	42.6 ± 7.3							
Horie et al. (21)	30.6 ± 9.4	<0.001	−0.01167	−0.05165	0.2260	0.817	10.45	3.22

New equation 2 = persons with obesity. FFM, fat-free mass; FM, fat mass; CCC, Concordance Correlation Coefficient; ρ, accuracy; C_b_, validity; PE, pure error; SEE, Standard Error of Estimate. Differences between predictive equations and reference method by paired t test.

The new regression models for predicting FFM (kg) are presented in Tables 5, 6. The performance of the models developed can be determined by the high coefficients of determination and low standard errors of the estimates, both in the equation for people with overweight or obesity, which explains 96% of the variability (r^2^ = 0.96; *p* < 0.001; SEE = 2.37 kg), and in the equation specifically developed for adults with obesity, which explains 94% of the variability (r^2^ = 0.94; *p* < 0.001; SEE = 2.57 kg). In both equations, the most important predictor was the resistance index; this variable alone explained 88% of the variability of the FFM obtained by the reference method (equation 1) (Table 5) and 85% in equation 2 (Table 6). For the two new equations developed in this study, the Mallows Cp value was close to the number of regressors, 8.6 for equation 1 (6 regressors) and 5.5 for equation 2 (5 regressors).

**Table 5:** Regression model for the prediction of FFM (kg) for persons with overweight or obesity.

Variables included in the model	Regression coefficient	r^2^	SEE	p-value	Collinearity statistics	
					Tolerance	VIF
Constant	−8.395			< 0.001		
Ht^2^/R (cm^2^/Ω)	+0.340	0.876^a^	3.896	< 0.001	0.197	5.075
Sex (male, 0; female,1)	−5.760	0.904^b^	3.433	< 0.001	0.324	3.087
Body mass (kg)	+0.222	0.939^c^	2.738	< 0.001	0.465	2.152
Age (years)	−0.041	0.950^d^	2.493	< 0.001	0.628	1.592
Height (cm)	+0.138	0.953^e^	2.420	< 0.001	0.269	3.721
PhA (°)	+0.700	0.955^f^	2.373	0.001	0.571	1.751

H^2^/R, resistance index; BMI: body mass index; PhA: phase angle; SEE: standard error of the estimate; VIF: variance inflation factor. Predictors: a. (Constant), Ht2/R; b. (Constant), Ht2/R, Sex; c. (Constant), Ht2/R, Sex, Weight; d. (Constant), Ht2/R, Sex, Weight, Age; e. (Constant), Ht2/R, Sex, Weight, Age, Height; f. (Constant), Ht2/R, Sex, Weight, Age, Height, PhA. The r^2^ change was significant for a, b, c, d, e, and f.

**Table 6:** Regression model for the prediction of FFM (kg) for persons with obesity.

Variables included in the model	Regression coefficient	r^2^	SEE	*p*-value	Collinearity statistics	
					Tolerance	VIF
Constant	−0.058			< 0.001		
Ht^2^/R (cm^2^/Ω)	+0.463	0.849^a^	4.121	< 0.001	0.210	4.771
Body mass (kg)	+0.278	0.889^b^	3.556	< 0.001	0.509	1.964
Sex (male, 0; female,1)	−5.150	0.926^c^	2.913	< 0.001	0.325	3.073
Reactance (Ω)	+0.115	0.940^d^	2.625	< 0.001	0.603	1.658
Age (years)	−0.049	0.943^e^	2.571	0.014	0.701	1.426

H^2^/R, resistance index; SEE: standard error of the estimate; VIF: variance inflation factor. Predictors: a. (Constant), Ht^2^/R; b. (Constant), Ht^2^/R, Body mass; c. (Constant), Ht^2^/R, Body mass, Sex; d. (Constant), Ht^2^/R, Body mass, Sex, Reactance; e. (Constant), Ht^2^/R, Body mass, Sex, Reactance, Age. The r^2^ change was significant for a, b, c, d, and e.

**Figure 3:**
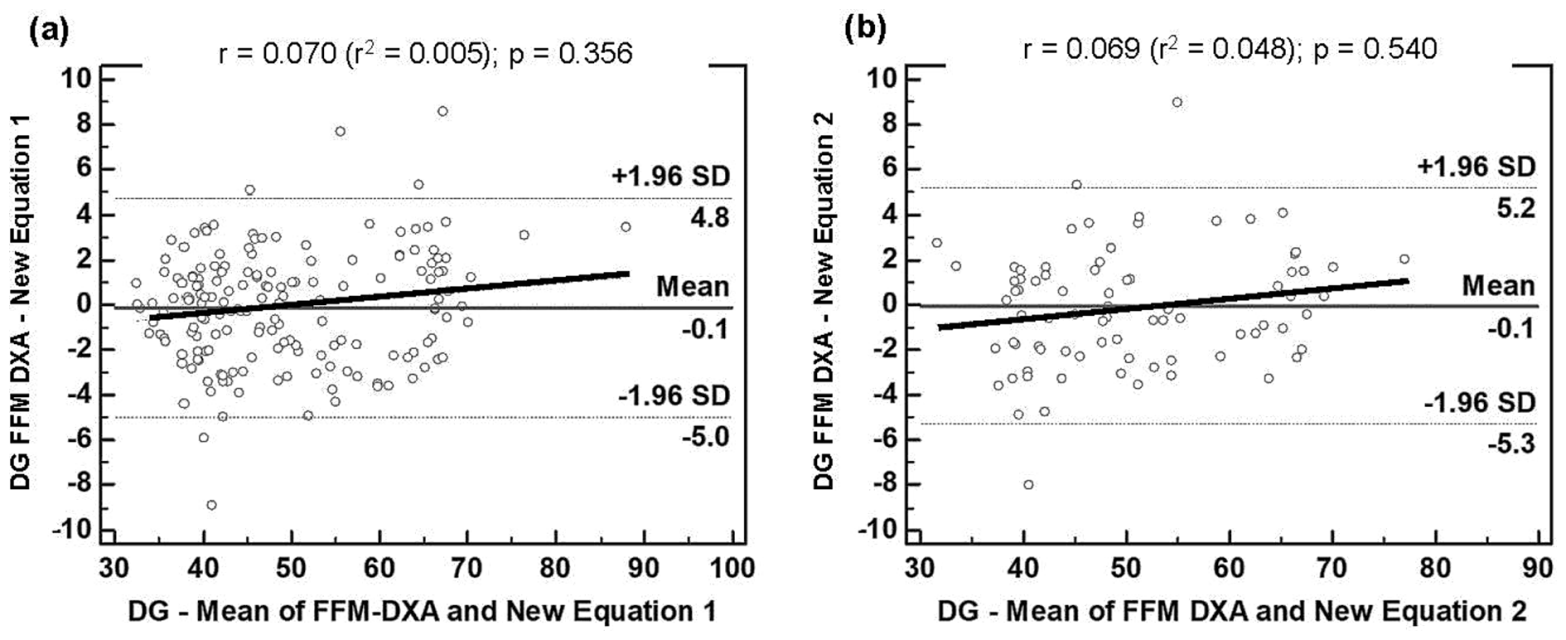
Bland–Altman plots for the concordance limits between values of DG determined by the reference method (DXA) and equations: **(A)** FFM derived in this study for people with overweight or obesity (Equation 1); **(B)**. FFM derived in this study for people with obesity (Equation 2). The dashed lines represent 95% limits of agreement. The proportional bias is represented by the slopping line. BIA, bioelectrical impedance analysis; DG, Development Group; DXA, dual-energy X-ray absorptiometry; Equation 1, first new regression equation developed to people with overweight or obesity; Equation 2, second new regression equation developed to people with obesity; FFM, fat-free mass; SD, standard deviation.

Figure 3 displays the agreement analysis for the development groups of the two new equations: (a) Equation 1, for people with overweight or obesity; and (b) Equation 2, for people with obesity. No association was found between the mean and difference of BIA and DXA (*p* > 0.05). The mean difference in the Bland–Altman plot was not different from zero for the two equations (*p* > 0.05); in addition, both showed acceptable LOA, indicating good agreement with the reference method.

The resulting prediction models included are presented below:

Equation 1: for people with overweight or obesity.
FFM = −8.395 + 0.340 Ht2/R - 5.760 Sex + 0.222 BM - 0,041 Age + 0.138 Ht + 0.700 PhA.
Equation 2: for people with obesity.
FFM = −0.058 + 0.463 Ht2/R - 5.730 Sex + 0.278 BM - 5.150 Sex + 0.115 Xc - 0.049 Age.
Sex: male = 0; female = 1.

Using predictive FFM, it is possible to calculate FM in kilograms by subtracting FFM from body mass (FM = BM − FFM). Then, it is also possible to calculate body fat percentage by the mathematical expression: FM% = (FM × 100)/BM.

Cross-validation showed excellent performance, both for Equation 1 (r^2^ = 0.96; *p* < 0.001; SEE = 2.04) and Equation 2 (r^2^ = 0.95; *p* < 0.001; SEE = 2.25), which can be observed in detail in Tables 3, 4, respectively.

In Figure 2, together with the other equations tested in this study, the agreement analysis of Equation 1 (a) and Equation 2 (e) is presented for the respective cross-validation groups. In line with what was found in the developing group for the new equations, there was no association between the mean and difference of BIA and DXA (*p* > 0.05), and the mean difference in the Bland–Altman plot was also not different from zero (p > 0.05). Good agreement with the reference method was also demonstrated by the LOA.

## Discussion

The equations developed and cross-validated in the present study are the first to provide an accurate FFM estimation in Brazilian adults with overweight/obesity and when applied specifically for those with obesity, as determined using DXA as a reference method. For both equations, all validity criteria were met, demonstrating excellent performance at the group level and acceptable individual accuracy.

In our study, we also tested the validity of three BIA equations used to estimate FFM for the general population (16, 18, 19), one for estimating the FFM of adults with obesity (17), and a second model for FM prediction in people with severe obesity (21); we hypothesized that better results would be observed when using population-specific equations that we developed compared to generalized equations or those developed for populations different from ours. As our hypothesis of low validity was confirmed for two of the three equations that were tested for people with overweight or obesity, and for the two specific equations tested for adults with obesity, we developed and cross-validated new equations for estimating FFM in the Brazilian population with overweight and obesity.

This interest arises from the growing prevalence of overweight and obesity worldwide (2, 32, 33), including in middle-income countries such as Brazil (3, 34), which indicates the need for simple and low-cost diagnostic techniques to promote prevention and treatment programs, as well as their follow-up. In this sense, the BIA has proven to be a valid technique, when properly used (13, 35, 36). A fundamental aspect of this good use concerns the availability of predictive equations that have been developed and validated for groups like those we intend to evaluate (9, 12, 22, 37).

There are few mathematical models developed to estimate FFM and body fat in overweight and obese people. To the best of our knowledge, the most used equations are those developed by Segal et al. (17) and Gray et al. (16), which are available in the BIA equipment software used in the present study, but there are other generalized equations used to evaluate overweight subjects and also unknown algorithms incorporated into the devices by the manufacturers. Therefore, we chose to test the validity of some of these equations in the present study sample.

Based on samples obtained from four different laboratories in the United States, using hydrostatic weighing as the criterion method, Segal et al. (17) developed an equation for each sample, involving 1,567 subjects (1,069 men, 498 women), aged 17 to 62 years, and with percentage of fat ranging between 3 and 56%, stratified by sex. Furthermore, the authors derived specific equations for people with obesity, indicating that the estimates obtained by bioelectrical impedance are reproducible and valid, and that the accuracy is increased by fatness-specific equations.

When testing the validity of the equation by Segal et al. (17) for estimating FFM in people with obesity in our sample, we found that it did not meet the validity criteria, as it showed a significant difference when compared to DXA (*p* = 0.001) and it demonstrated high LOA, thus reducing its applicability at the individual level. It is possible that these high LOA values are associated with differences between the characteristics of the groups from which the equation was derived and our sample, given the dissimilar ethnicity and sociodemographic characteristics of these two populations. Thus, all these factors must be carefully considered when choosing a BIA-predictive equation (38, 39).

Also using hydrostatic weighing as the gold standard, Gray et al. (16) tested the validity of the equations proposed by Segal et al. (17) in a sample of 25 men and 62 women, who were between 19 and 74 years of age and varied widely in body composition, with body fat ranging from 8.8 to 59.0%. The authors found that the previously developed equations tended to overestimate body fat, especially in the subjects with higher levels of adiposity. Thus, they derived new mathematical models for their data, in which variables for body mass, height squared, resistance and age were included.

We also tested the validity of the equations derived by Gray et al. (16) in our sample, verifying that there were no significant differences when the FFM results were compared with those obtained by DXA (*p* > 0.05). However, the only reasonable LOA (−6.4 to 6.3) demonstrated that these equations were not the most appropriate for estimating the FFM at the individual level in the present study. It should be noted that the BIA equipment used in the present study (Sanny^®^) only provided the equations proposed by Segal et al. (17) and Gray et al. (16) for the evaluation of individuals with obesity, indicating the need for new equations in the device’s software for use in this population group.

Thus, we tested the validity of two more generalized equations for particular age group and nutritional status classifications. In the first equation, which was developed by Kyle et al. (18) using DXA as a reference method in a sample of 343 healthy white subjects, aged 20 to 94 years and with BMI varying between 17.0 and 33.8 kg/m^2^, accuracy in the cross-validation was observed. However, when we tested the validity of this equation for estimating FFM in the cross-validation group used to developed equations for individuals with overweight/obesity, we found that it overestimated the FFM obtained by DXA (*p* < 0.001). This fact can be explained by the different ethnic backgrounds between the Swiss population (Caucasian) and the Brazilian population, with great ethnic mixing (23). Several previous studies have demonstrated differences in the proportions and densities of body components between different races and ethnicities (40–42). Another issue is that differences in fat-free body density compartments have been observed between races and ethnicities, which may lead to differences in total body density and therefore in the estimation of body composition by different techniques (43, 44).

In the model developed by Macias et al. (19), which used ADP as the reference method in a sample of 155 Mexican men and women, aged 20 to 50 years and with BMI varying between 17.8 and 35.5 kg/m^2^, the authors concluded that the equation was accurate, precise and free of bias for the assessment of body composition and nutritional status in populations similar in physical and anthropometric characteristics to those of the population of origin of the equation. However, in our cross-validation sample (individuals with overweight and obesity), the Mexican equation overestimated the mean values of FFM compared to the mean obtained by DXA (*p* = 0.005), showing a proportional bias at the individual level and poor agreement with the reference method (LOA: −7.1 to 4.1).

In Brazil, Horie et al. (21), developed an equation to estimate body fat in people with severe obesity. Using ADP as a criterion in a sample of 119 subjects (83 female and 36 male) aged 18 to 62 years and with BMI varying between 34.4 and 59.6 kg/m^2^, the authors observed a better accuracy in relation to the values obtained by the equation of the software of the equipment (QuadScan 4,000, Bodystat Ltd., Douglas, UK). Although our sample is not composed of individuals with severe obesity, we tested its validity because it was developed for people in the Brazilian population with obesity. However, the performance of the model was the poorest of those tested, with the estimated FM significantly underestimated compared to that obtained by DXA (*p* < 0.001), in addition to having high PE (10.45), high proportional bias and wide LOA (−1.9 to 38.7). These results are thought to be explained by the fact that the BMI of our subjects was lower than 40 kg/m^2^.

It is recognized that the use of predictive equations developed and cross-validated in groups with characteristics similar to those of the original population we intend to evaluate can reduce discrepancies in the results obtained (10, 45, 46). However, the available equipment does not always provide equations that cover different population groups. For this reason, the development of mathematical models for the growing group of people with overweight and obesity is very important (47).

One study suggests that BIA equipment should provide equations developed for different population groups in their software, which could be chosen by the health professional according to the characteristics of the people to be evaluated (9). However, if the equipment does not provide this possibility, then the results of resistance, reactance, and other raw BIA data, can be used in a theoretically valid equation for the individuals or groups being evaluated.

A strength of this study was the development of the first equations specifically derived for people with overweight or obesity in Brazil, using DXA as the reference method. In addition to low VIF and excellent tolerance values, these equations showed a high coefficient of determination and good LOA in relation to DXA, providing valid estimations that can be potentially used to track FFM changes resulting from diet and/or exercise-induced weight loss or FFM gains due to resistance exercise training programs (47, 48).

However, some limitations must also be addressed. This study included a sample of people with overweight or obesity from only one region of the country, and ethnicity was not reported. Hydration status was not assessed using a measure such as urine specific gravity, and it is not possible to know whether euhydration status was being maintained. Nevertheless, a recent study aiming to compare the body water compartments and hydration status of athletes with different habitual water intakes (low vs. high water drinkers, with higher and lower urine specific gravity values, respectively) found no differences in total body water (TBW) and FFM hydration between groups for both sexes (49). The period of the menstrual cycle in which the women were on the day of data collection was not controlled, but some recent studies have shown that bioimpedance can be performed without significant differences in body composition results, throughout the different periods of the menstrual cycle (50–52). Although the sample size was adequate for Equation 1, the limited sample size for Equation 2 may limit its applicability. Still, for both equations, the validation and cross-validation criteria were met. Other studies carried out in Brazil for the development of predictive equations by BIA also used samples without reporting or distinguishing ethnic differences (21, 23), which suggests the need to validate the newly proposed models in other regions of the country and with subjects of different ethnic origins. Additional studies should also be conducted to test the accuracy of the proposed equations in tracking FFM changes as a result of weight management programs. Another important issue concerns the criterion method that was employed, which was the DXA; however, its validity against the four-compartment model (4C) is acceptable in adults. Indeed, the 4C model is the most appropriate reference method for evaluating FM and FFM at the molecular level, as it allows the quantification of the main FFM components by reducing major assumptions (53), and BIA-developed models have been derived in an Hispanic population for older adults with excess adiposity (20). However, due to the complexity of the technique, and the associated propagation of measurement error that may limit its use, the utility of DXA for deriving BIA equations has been widely accepted (22, 54).

## Conclusion

Based on the performance of the BIA models, the equations developed in this study were valid for estimating FFM, whereas the equations developed in other countries lack accuracy when applied to Brazilian individuals with overweight/obesity. Thus, these new BIA-derived models can be considered good alternatives for assessing body composition in a Brazilian population living with overweight or obesity.

## Data Availability

The raw data supporting the conclusions of this article will be made available by the authors, without undue reservation.
